# Anterior communicating aneurysm clipping: How I do it

**DOI:** 10.1007/s00701-025-06440-8

**Published:** 2025-01-27

**Authors:** Bruno Vernile, Paolo Palmisciano, Sudhakar Vadivelu, Mario Zuccarello

**Affiliations:** 1 Division of Pediatric Neurosurgery, Department of Neurosurgery, Cincinnati Children’s Hospital Medical Center, University of Cincinnati College of Medicine, Cincinnati, OH USA; 2 Department of Neurosurgery, University of California, Davis, Sacramento, CA USA

**Keywords:** Anterior communicating aneurysm, Aneurysm clipping, Cerebral aneurysm, Aurgical approach

## Abstract

Up to 40% of intracranial aneurysms arise from the anterior cerebral artery and anterior communicating artery (ACA-ACoA) complex. The vast variability of vessel anomalies and the surrounding critical structures correlate with severe morbidity and mortality rates in case of rupture. In the era of cutting-edge advantages of endovascular procedures, surgical expertise is reducing. This article describes our institutional surgical technique in managing ACoA aneurysms, focusing on anatomical variants, approach selection, and technical intraoperative nuances.

**Supplementary information:**

The online version contains supplementary material available at 10.1007/s00701-025-06440-8.

## Introduction

The anterior communicating complex (ACC) is defined as the combination of the distal A1 segments of the anterior cerebral arteries (ACAs), the anterior communicating artery (ACoA), and the proximal A2 segment of the ACAs. Around 40% of intracranial aneurysms arise from the ACC, with an increased tendency to rupture and related morbidity and mortality [5]. ACC closely surrounds the optic chiasm, optic nerves, and perforating arterial branches that, if injured directly intraoperatively or indirectly by postoperative edema or vasospasm, could result in severe neurologic sequelae. Additionally, the ACC anatomy can vary among individuals. In this article, we describe our institutional surgical management of ACA-ACoA complex aneurysms.

### Relevant surgical anatomy

Up to 40–50% of cases may show a variant ACC, with the most frequent being unilateral hypoplasia of A1 segment (31%), especially of the right side, correlated with a higher incidence of ACoA aneurysms [7].

Regarding ACA2 variants, ACA2 fusion is the most frequent anomaly (9%); rare is the azygos-shaped A2 in case of complete or partial regression of the ACAs. [2, 7]

ACoA may be duplicate and triplicate up to 30% and 10% of cases, respectively [6].

### Complex regional anatomy

The ACoA uncommonly runs horizontally between two parallel ACAs (only 18%). In most cases, the left ACA runs anteriorly to the right ACA (48%), and less frequently posteriorly to it (34%), leading to an oblique or sagittal course of the ACoA [7].

Perforating arteries arise from the superior and dorsal portion of the ACA1 (MLAs) and from the superior and posterior aspect of the ACoA. Injury of those arteries leads to motor, sensory, and visual disturbances.

Three branches originate from the ACA2: *recurrent artery of Hubner* (RAH), *orbitofrontal artery* (OFA), and *frontopolar artery* (FPA). Injury to one of these arteries can lead to motor, sensory, and cognitive dysfunction [3, 9].

### Surgical technique

#### Preoperative assessment

A CT angiography is beneficial to rule out an aneurysm calcification that would make aneurysm clipping complex and dangerous. Digital subtraction angiography (DSA) provides more detailed features of the aneurysm allowing the choice of the optimal approach. MRI is usually reserved for unruptured aneurysms to evaluate their relationships with surrounding structures.

Pre-operative imaging is necessary for evaluating:

1. The extent of the circle of Willis and ACA1 dominance, as the ACoA may supply a large portion of the contralateral hemisphere.
2. Aneurysm projection, height, width, branching vessels, and adherence to surrounding structures.
3. The ACC height from the skull base bony anatomy, which dictates the complexity of the dissection:

 

a < 10 mm height from the ACP is considered low, with the dissection being relatively simple due to direct visualization of the ACC with minimal Sylvian fissure dissection and frontal lobe retraction;
b > 12 mm height from the anterior clinoid is considered high, with potentially extensive frontal lobe retraction combined and sylvian/interhemispheric fissures dissection, adding additional difficulty to the procedure
c  ≥ 15 mm height superiorly projecting aneurysms are very difficult to treat surgically

4. Development of the anterior communicating artery, as it may dictate the side of the approach.

If the AcoA-ACA junction is located significantly higher on the right side than the AcoA-ACA junction on the left, it is easier to apply a clip parallel to the ACoA artery when approaching the aneurysm from the left because of a lower angle related to the interhemispheric fissure.

5. Size of the aneurysm neck: small or large neck.
6. Calcifications of the aneurysm.

#### Pre-procedure plan

- Turn the head no more than 15 degrees toward your approach’s contralateral side.
- Intraoperative neuromonitoring (IONM) to monitor neurological function is routinely used and in case of temporary clipping when a SBP is increased of 20% from the baseline is recommended. (Ref)
- Obtain maximal cerebrospinal fluid (CSF) release by putting an external ventricular drain, lumbar drain, and performing a lamina terminalis fenestration, routinely in case of ruptured aneurysms.
- Intraoperative mannitol is not routinely used at our institution.

#### Side of approach

The choice of the side of approach depends on the dominance of the A1 or if an intraparenchymal hematoma needs to be evacuated. Right approach may be more favorable for right-handed surgeons, regardless of A1 dominance, except in situations of an angled ACoA, as mentioned above.

#### Craniotomy

Treating ACoA aneurysms by pterional or supra-/trans-orbital approaches is preferred at our institution^1^. The transorbital approach provides a significant benefit over the pterional because it allows a steeper angle to approach the interhemispheric fissure, useful in unruptured aneurysms. In case of a ruptured aneurysm, an orbito-pterional bone-flap is preferred [1]; in case of unruptured aneurysms, a standard pterional approach is performed [10].

#### Arachnoid dissection

**Fig. 1:**
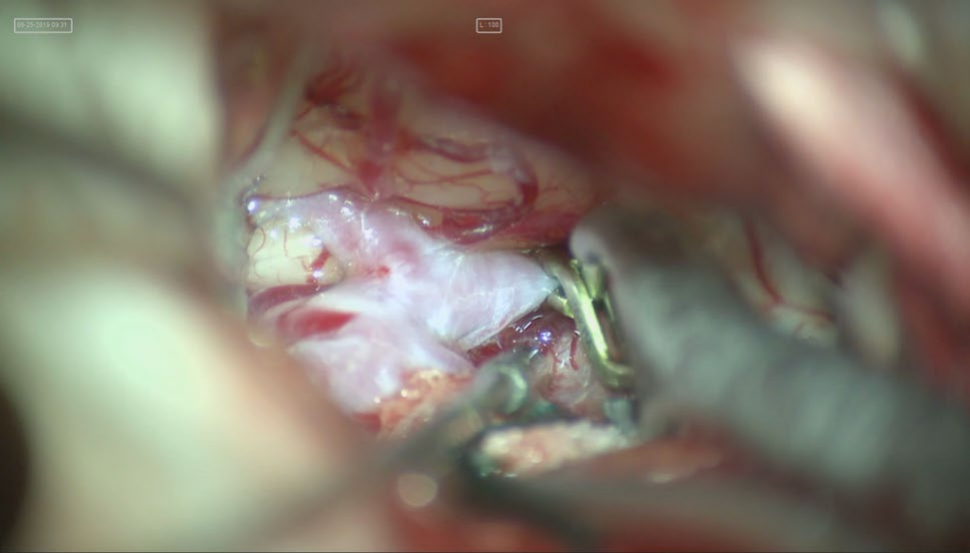
Temporary clip of the contralateral A1

**Fig. 2:**
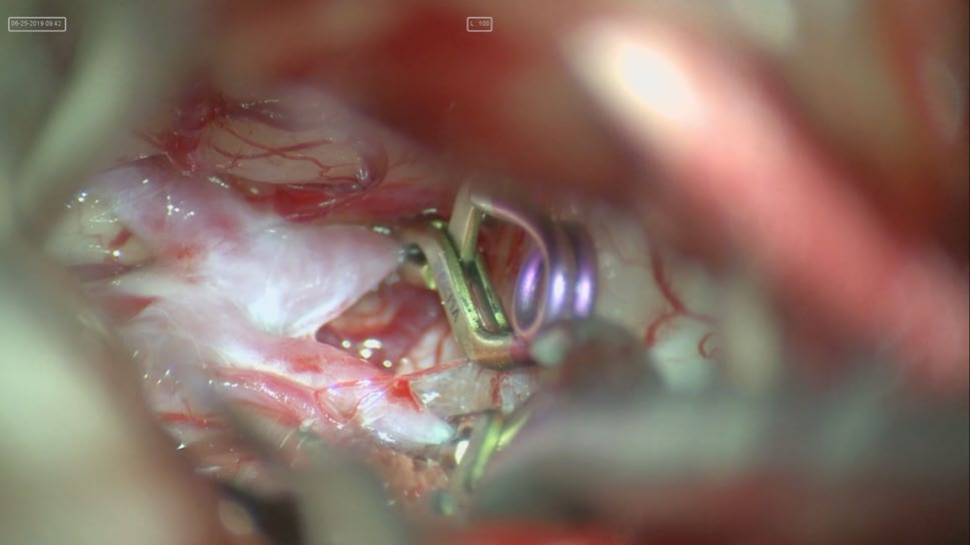
Temporary clip of the homolateral A1

- Start the dissection from the optic nerve.
- Dissect the ophthalmic segment of the carotid.
- Travel back to the chiasm and then open the Lilliquist membrane for more CSF egress.
- Identify ipsilateral A1.
- Travel down to the contralateral optic nerve to identify contralateral A1.
- Dissect the interhemispheric fissure to identify the distal A2 segments.
- 5 mm temporary clip on the contralateral A1 segment (Fig. 1).
- Further dissection of the interhemispheric fissure.
- After identification of the neck of the aneurysm, place a temporary clip on the ipsilateral A1 for complete arrest prior to permanent clip application (Fig. 2).

For high-riding aneurysms or in the case of co-dominant A1s, in addition to orbitotomy, partial resection of the gyrus rectus may be needed to see the superior portion of the neck.

#### AcoA aneurysm type and clip selection

Apply the clip parallel to the ACoA to preserve the parent vessel.

**Fig. 3:**
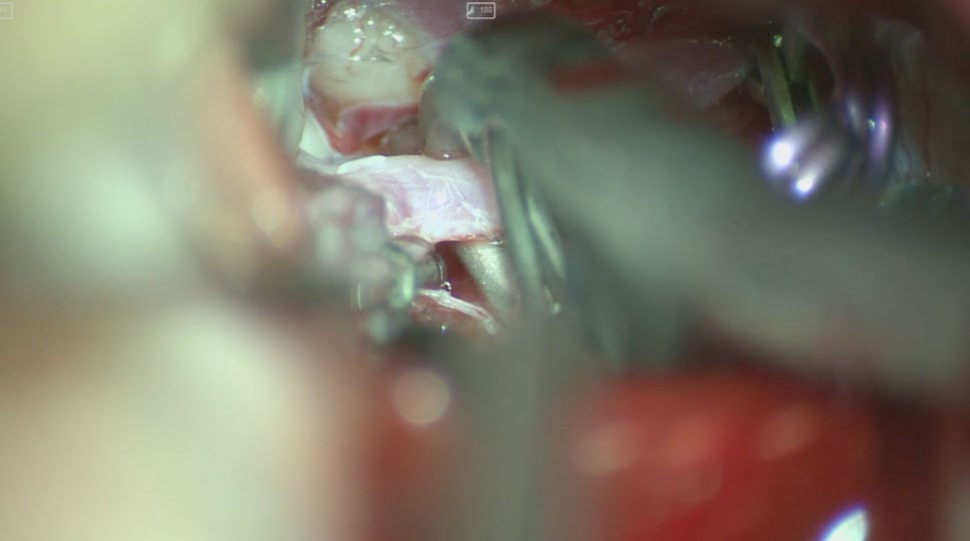
ACoA aneurysm clipping with a fenestrated clip

- Anteriorly projecting aneurysm: use a straight clip parallel to the base of the aneurysm;
- Superiorly projecting aneurysm: straight 3 mm fenestrated clip, because the length of the ACoA is approximately 3 mm, therefore it should be safe and not occlude the contralateral ACA (Fig. 3);
- Posteriorly projecting aneurysm: all of the perforators are strapped on the inferior part of the dome of the aneurysm with subsequent increased risk of ischemic events. All perforators should be dissected from the neck and dome of the aneurysm before clipping.
- Inferiorly projecting aneurysm: use a straight clip and do not care about perforators.

#### Post-operative follow-up

- Euvolemia, BP control and daily TCDs.
- DSA in case of evidence of vasospasm.

### Surgical indications

- Superior/inferior projecting aneurysms.
- Difficult endovascular approach.
  - Dome-to-neck ratio less than 2.
  - Too small to coil (less than 3 mm).
  - Wide neck non amenable to balloon remodeling.
  - Contraindication to anti-platelet meds.
- Complex morphology.
  - Partially thrombosed.
  - Previous failed coiling.
- Multiple anterior circulation aneurysms.
- Young age.
- Intraparenchymal hemorrhage.

### Limitations

- Aneurysm Dome Projection.
- Posterior dome projection risk factor for ischemic complications.
- Calcified Aneurysms.
- Increasing age (> 65 years risk of poor outcome increases 11-fold).

### How to avoid complications

1. Always use temporary clip in ruptured aneurysms before permanent clipping
2. Identify structures that provide complications:
  1. No calcifications in aneurysm neck.
  2. Recurrent artery of Heubner.
3. Appropriate clip selection before starting clipping.
4. Avoid trapping the AcoA because may lead to loss of all perforators.

### Specific information to give to patients about surgery and potential risks

Make the patients aware about potential complications:

- ischemic complications in frontal infarction with significant motor, sensory, and cognitive deficits, and memory loss.
- cerebrospinal fluid leakage with need for further treatment.
- skin- and wound-healing complications.

### Summary of key points

- Knowledge of complex regional anatomy
- Knowledge of complex vascular anatomy
- Understanding of aneurysm and surrounding vessels anatomy on x-rays
- Proper surgical approach (unruptured vs. ruptured aneurysms, multiple aneurysms, size, shape, direction)
- Maximal skull exposure
- Maximal brain relaxation (lamina terminalis)
- Maximal blood vessel visualization allows perfect clip application and vessel inspection

## Supplementary Information

Below is the link to the electronic supplementary material.

Supplementary file1 (MP4 24618 KB)

## Data Availability

No datasets were generated or analysed during the current study.

## Abbreviations

*ACoA*: Anterior Communication Artery
*ACA*: Anterior Cerebral Artery
*ACC*: ACoA-ACA complex
*RAH*: Recurrent Artery of Huebner
*MLAs*: Medial lenticulostriate perforating arteries
*OFA*: Orbitofrontal artery
*FPA*: Frontopolar artery
*CTA*: Computer tomography angiography
*DSA*: Digital subtraction angiography
*ACP*: Anterior clinoid process
*TCD*: Trans-cranial-doppler
*BP*: Blood pressure
*SBP*: Systolic blood pressure
